# Self-Healing Products of Cement Pastes with Supplementary Cementitious Materials, Calcium Sulfoaluminate and Crystalline Admixtures

**DOI:** 10.3390/ma14237201

**Published:** 2021-11-25

**Authors:** Byoungsun Park, Young-Cheol Choi

**Affiliations:** 1 Department of Environmental Systems Engineering, Sejong Campus, Korea University, Sejong-si 30019, Korea; bspark0927@korea.ac.kr; 2 Department of Civil and Environmental Engineering, Gachon University, Seongnam-si 13120, Korea

**Keywords:** self-healing concrete, self-healing products, further hydration, phase composition, supplementary cementitious materials

## Abstract

The phase composition of self-healing products generated in cracks affects self-healing performance. This study investigated the self-healing products of cementitious materials using supplementary cementitious materials (SCMs), a calcium sulfoaluminate (CSA) expansion agent, and crystalline additives (CAs). Ground-granulated blast-furnace slag (GGBFS), fly ash (FA), and silica fume (SF) were used as SCMs, and anhydrite, Na_2_SO_4_, Na_2_CO_3_, and MgCO_3_ were used as crystalline additives (CAs). An artificial crack method was used to collect the self-healing products in the crack of the paste. The phase composition of the self-healing products was analyzed through X-ray diffraction (XRD)/Rietveld refinements and thermogravimetry/differential thermogravimetry (TG/DTG) analysis, and their morphology and ion concentration were examined through scanning electron microscopy with energy dispersive spectroscopy (SEM–EDS). From the results, the main compound of self-healing products was found to be calcite. GGBFS and FA decreased the content of portlandite, and the use of CAs led to the formation of alkali sulfate and alkali carbonate. The SEM–EDS analysis results showed that when GGBFS and FA were used, a large proportion of the self-healing products contained C-S-H and C-A-H, and the use of CSA led to the formation of monosulfate and ettringite.

## 1. Introduction

Concrete is the most widely used construction material worldwide, owing to its high compressive strength and low cost; however, it has a high risk of cracking because of hydration, heat and contraction resulting from its relatively low tensile strength [1,2,3,4]. Cracks generated in concrete act as passages for moisture and harmful ions, thus degrading the durability of concrete structures [5,6]. Therefore, crack management in concrete is one of the most important aspects in the lifecycle of structures, including construction and maintenance. In terms of crack management, researchers have developed a self-healing concrete technology that can prevent durability reduction by healing cracks in concrete structures owing to the inherent properties of the structures [7,8,9,10,11,12,13,14,15].

Cementitious materials have a natural characteristic in that cracks of certain widths can be self-healed, in a process called autogenous healing [16,17,18]. During the autogenous healing of cracks, unreacted cement particles on the crack surface react with water penetrating through the cracks, which are then filled by the products of further hydration [19,20]. Therefore, the autogenous healing characteristic of concrete depends on the characteristics of the binder. Recently, many studies have been conducted to improve crack healing performance through autogenous healing using mineral additives [21,22,23,24,25,26,27,28,29,30,31].

Tittelboom et al. [21] evaluated the autogenous healing characteristic in terms of the type and mixture proportion of supplementary cementitious materials (SCMs) using water flow tests and isothermal calorimetry. They reported an improvement in the autogenous healing performance after adding ground granulated blast-furnace slag (GGBFS) and fly ash (FA). In addition, they analyzed the healing products in the surface cracks and reported that the precipitation of CaCO_3_ was the most important factor determining autogenous healing performance. Termkhajornkit et al. [22] generated microcracks by autogenous shrinkage in FA–cement systems and investigated the recovery of physical performance through self-healing. They induced cracks by autogenous shrinkage and measured the compressive strength and chlorine ion diffusion coefficient with respect to the mixture proportion of FA. They noted an increase in the long-term compressive strength and chlorine ion diffusion coefficient with an increasing substitution rate of FA. They reported that FA improved self-healing performance through the pozzolanic reaction of unreacted FA particles. Sisomphon et al. [23] evaluated the autogenous healing performance of cementitious materials mixed with a calcium sulfoaluminate (CSA) expansion agent and crystalline additives (CAs). They evaluated the autogenous healing performance by measuring the crack width reduction due to autogenous healing using an optical microscope. When ordinary Portland cement (OPC) was replaced with 10% CSA and 1.5% CAs, 400-μm cracks could be healed within 28 days. In particular, they claimed that CSA and CA increased the content of Ca^2+^ ions in the cracks by diffusing through the crack surface, which promoted the precipitation of CaCO_3_ and improved the autogenous healing performance. Jaroenratanapirom and Sahamitmongkol [24] evaluated the autogenous healing performance of mortar specimens containing expansion agents and CAs. They reported that CA exhibited a high healing performance for cracks with widths in the range of 0–0.05 mm; however, the performance decreased when increasing the crack width. Roig-Flores et al. [25] and Azarsa et al. [26] investigated the autogenous healing characteristics of concrete containing CAs under various binder and environmental conditions. They experimentally verified that water is indispensable for autogenous healing of concrete containing CAs. Some researchers used superabsorbent polymers (SAPs) to improve autogenous healing performance in cementitious materials [32,33,34,35]. They reported that SAPs improved the autogenous healing performance of cementitious materials. However, they focused on the self-sealing properties of SAPs, not on self-healing products themselves.

A literature review shows that most of the existing studies simply evaluated the autogenous healing performance of cement composites and analyzed the self-healing products formed in surface cracks. Huang et al. [36] analyzed self-healing products generated in the cracks of a paste containing GGBFS. They reported that ettringite formed in the cracks by autogenous healing in a paste specimen containing GGBFS. Moreover, they also reported that the self-healing performance of a paste specimen containing GGBFS is higher than that of a normal paste specimen. Li et al. [37] investigated the self-healing products of mortar specimens containing GGBFS and CAs using X-ray diffraction (XRD) and scanning electron microscopy with energy dispersive spectroscopy (SEM–EDS). They reported that the main component of self-healing products is calcite. Park and Choi [38] examined the relationship between self-healing products and the self-healing performance of cementitious materials with GGBFS, CSA, Na_2_SO_4_ and Al_2_(SO_4_)_3_. They reported that the phase composition of self-healing products affects the self-healing performance of cementitious materials. However, their research was limited to investigating the effect of GGBFS and some CAs on self-healing properties. Studies of self-healing products generated in the cracks according to various types of SCMs and CAs are particularly lacking. A self-healing concrete containing various types of SCMs and CAs could exhibit improved autogenous healing performance by controlling the self-healing products through further hydration. Hence, it is necessary to investigate the phase composition of self-healing products according to types of SCMs and CAs to predict self-healing performance.

Accordingly, this study investigated the chemical and phase composition of self-healing products generated inside the cracks of cementitious materials made using SCMs, a CSA expansion agent, and crystalline additives (CAs). SCMs have a lower reaction rate than OPC, so a greater amount of SCMs remain in the matrix compared to OPC at the same age. For this reason, SCMs can enhance self-healing performance through further hydration. CSA expansion agents can efficiently fill cracks by generating expandable hydrates such as ettringite. In this study, two types of CA were used: CA containing SO_4_^2−^ and CA containing CO_3_^2−^. CA containing SO_4_^2−^ was used to induce the formation of ettringite, and CA containing CO_3_^2−^ was used to induce the formation of calcite. GGBFS, FA, and silica fume (SF) were used as SCMs, and anhydrite, Na_2_SO_4_, Na_2_CO_3_, and MgCO_3_ were used as CAs. To analyze the self-healing products generated in the cracks, artificial cracks of a constant width were produced by overlapping slice specimens. Subsequently, the products generated in the artificial cracks were collected. The self-healing products were analyzed using XRD/Rietveld refinements, thermogravimetry/differential thermogravimetry (TG/DTG) analysis, and SEM–EDS.

## 2. Materials and Experiments

### 2.1. Materials

This study used OPC, GGBFS, FA, SF, and CSA as raw materials. Table 1 lists the chemical compositions and physical properties of the raw materials. 

**Figure 1:**
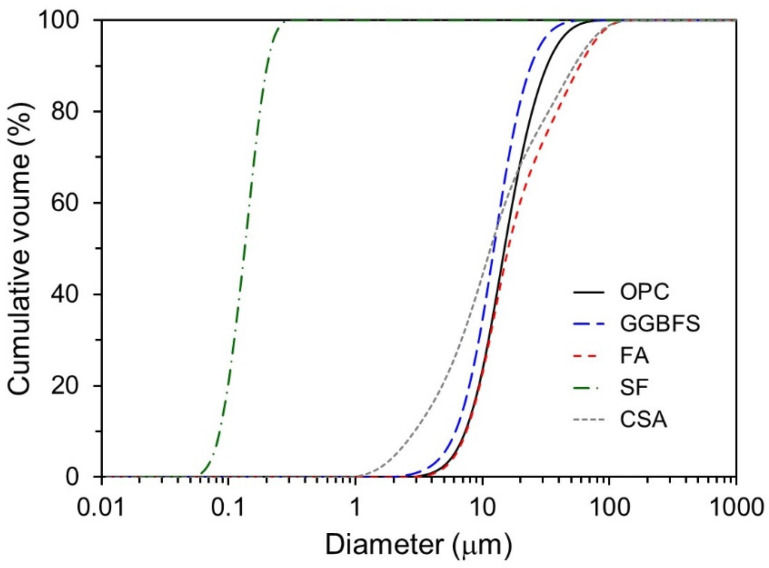
Cumulative volume of particle size distributions of raw materials.

Figure 1 shows the particle size distributions of the raw materials measured by laser diffraction scattering (LA-960, HORIBA). OPC, GGBFS, and SF exhibited monomodal distributions, whereas CSA and FA both exhibited a bimodal distribution.

**Figure 2:**
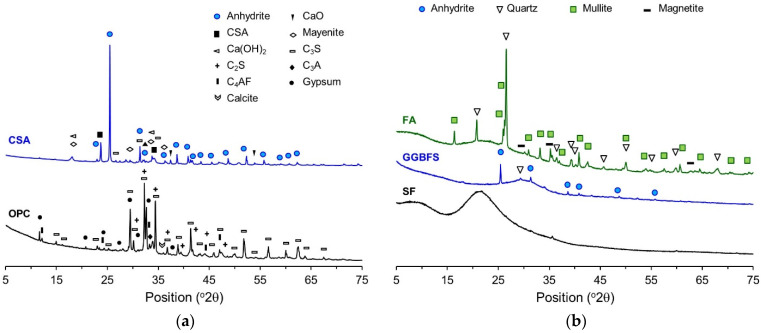
X-ray diffraction (XRD) pattern of raw materials. (**a**) Ordinary Portland cement (OPC), Calcium sulfoaluminate (CSA). (**b**) Fly ash (FA), Ground granulated blast-furnace slag (GGBFS), Silica fume (SF).

XRD/Rietveld refinement was performed to calculate the contents of the constituent minerals. Figure 2 shows the XRD patterns of the raw materials. XRD/Rietveld refinement shows that the main constituent minerals in the OPC were C_3_S, C_2_S, C_3_A, and C_4_AF with contents of 62.0, 16.1, 2.5, and 12.1%, respectively. In addition, the OPC contains 2.6% gypsum and 4.5% limestone powder as a mineral admixture. Moreover, the XRD analysis result shows that the GGBFS is largely made of an amorphous phase, accounting for 95%, and the crystalline phase is composed of anhydrite (2.9%) and quartz (2.1%). The SF does not exhibit any peaks in the XRD patterns, and seems to be mostly made of amorphous phase. The FA is composed of 74.4% amorphous phase and 25.6% crystalline phase. The crystalline phase is composed of mullite, quartz, and magnetite, accounting for 13.9, 11.3, and 0.4%, respectively. The CSA is mostly composed of a crystalline phase, and the major constituent minerals are gypsum, calcium sulfoaluminate, and calcium hydroxide, accounting for 48.6, 28.0, and 16.6%, respectively.

### 2.2. Mixture Proportions

Table 2 lists the mixture proportions of the paste specimens. In this study, the water-to-binder (W/B) ratio was fixed at 0.4. To examine the effects of the type of SCMs on autogenous healing, specimens were fabricated with different types of SCMs and replacement ratios. Furthermore, to improve autogenous healing performance, specimens were fabricated using crystalline additives such as anhydrite, Na_2_SO_4_, Na_2_CO_3_, and MgCO_3_.

### 2.3. Test Methods

To analyze the self-healing products generated in concrete cracks, the self-healing products must be separated from the cracks. Since concrete cracks are typically induced in irregular shapes, it is difficult to separate the existing cement matrix and the autogenous healing product. In this study, self-healing products were collected from artificial cracks using a method suggested by Huang et al. [36]. The detailed experimental method can be found in previous paper [38]. 

**Figure 3:**
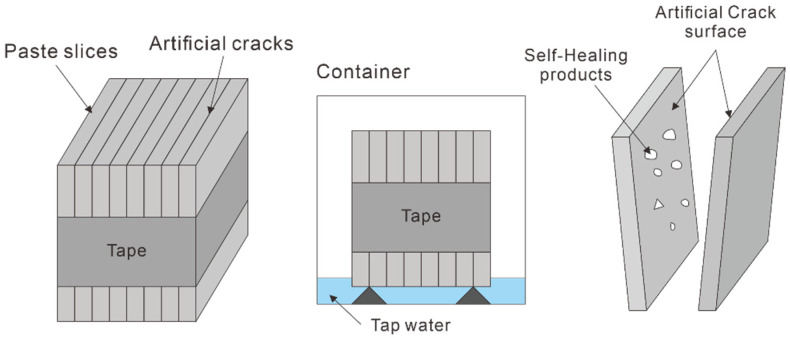
Experimental diagram of the artificial crack method [36].

To generate artificial cracks, 32 paste slice specimens with dimensions of 100 mm × 100 mm × 10 mm were fabricated. These specimens were cured in a chamber at 20 ± 1 °C and 100% RH for 24 h. After 24 h, the specimens were demolded and cured in a water container at 20 ± 1 °C until the age of seven days. At the age of seven days, they were dried in an oven at 40 ± 1 °C for 4 h. The surfaces of the dried specimens were polished to expose the unreacted materials inside them with a polishing depth of 0.1 mm or more. A 100 μm-thick polyester film was laid on the interfaces of each polished specimen and fixed using a tape. The bundle specimens in which artificial cracks were generated were placed on a 20-mm-thick spacer, and tap water was poured, such that 5 mm of the bottom of the specimen would contact the water. Figure 3 shows experimental diagram of the artificial crack method

All the bundle specimens were separated after immersion for seven days. Immediately after separating them, the self-healing products were collected from the artificial cracks using a plastic putter. The self-healing products were dried in an oven at 40 ± 1 °C for 24 h, and the hydration of self-healing products was stopped using isopropanol before conducting a chemical and phase composition analysis (XRD, TG/DTG, and SEM-EDS). After the self-healing products were immersed in isopropanol for 24 h with a solid/liquid ratio of 1/50, hydration was stopped by replacing the solvent through vacuum. Subsequently, the self-healing products were kept in the isopropanol in a chamber at 23 ± 1 °C until the chemical composition analysis was performed. To examine the chemical composition and morphology, powder samples smaller than 75 μm were prepared by grinding the products.

In this study, the phase composition of self-healing products was investigated using XRD/Rietveld refinement and TG/DTG analysis. A PANalytical X’Pert Pro MRD diffractometer (Malvern Panalytical, Malvern, UK) equipped with a X’Celerator detector was used for the XRD measurement. The diffractometer scan was performed at 2θ values ranging from 10 to 60° with a step size of 0.04° and a counting time of 2 s. A Rietveld quantitative phase analysis was performed to quantify the crystalline mass using the X’Pert HighScore Plus (PANalytical) software (Malvern Panalytical, Malvern, UK). For the internal standard material, 10% corundum was added. The contents of portlandite and calcite in the self-healing products were analyzed using thermogravimetry/derivative thermogravimetry (TG/DTG). For the TG/DTG analysis, a Thermo Plus EVO II (Rigaku, Tokyo, Japan) was used. The TG/DTG analysis was performed while heating the specimens at a rate of 10 °C/min in a nitrogen atmosphere of 300 mL/min, and the measurement temperature range was 20–1000 °C.

The morphology and chemical composition of self-healing products were investigated using a field emission scanning electron microscope (FE-SEM, HITACHI SU 8220, Tokyo, Japan) at 15 kV and a working distance (WD) of 15.8 mm.

## 3. Results and Discussion

### 3.1. Quantitative Analysis of Hydration Products in Cracks

**Figure 4:**
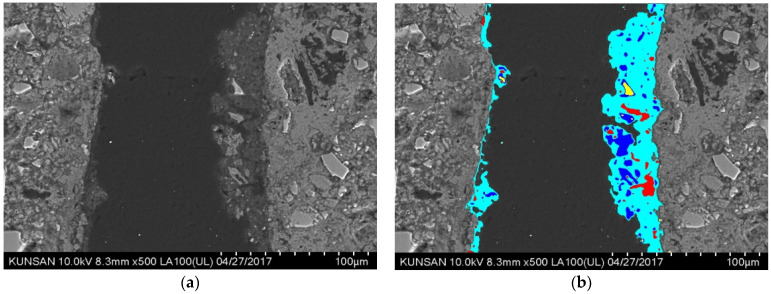
Artificial crack and self-healing product (G40NS5). (**a**) BSE image. (**b**) Self-healing product.

Figure 4a shows the backscatter electron (BSE) images of the self-healing products generated in an artificial crack of G40NS5. The product was generated on the crack surface through further hydration of the unreacted material on the crack surface between approximately 100-µm artificial cracks. The black and white image in Figure 4b shows the existing paste, and the colored areas represent the additional self-healing products generated during the seven-day autogenous healing period. The self-healing products were collected carefully using a plastic putter to prevent wearing of the existing cement matrix. The quantity of the collected sample was measured and its composition was analyzed.

**Figure 5:**
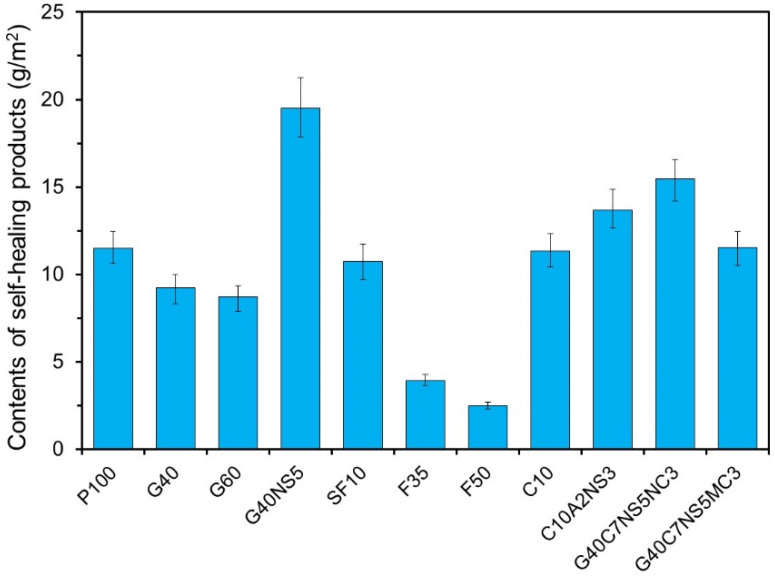
Contents of self-healing products in artificial cracks.

Figure 5 shows the quantities of the self-healing products generated in the artificial cracks. The Y-axis indicates the contents of self-healing products generated in the cracks shown in units of amount per crack surface area (g/m^2^). The quantities of the products were indicated by the content of the product per crack surface. The quantities of the products were measured after the content of the self-healing product of P100 was 11.49 g/m^2^. The graph shows that in the case of specimens containing GGBFS and FA, the contents of self-healing products decreased regardless of the replacement ratio. For FA, the content decreased to less than half of the P100. This was because as the replacement ratio of FA increased, the OPC content decreased, and the amount of Ca(OH)_2_ generated by the hydration of OPC decreased. Subsequently, the pozzolanic reaction decreased due to the reduction of Ca(OH)_2_ [39]. The GGBFS also showed a reduction in the self-healing products with an increasing replacement ratio. This is inconsistent with an existing finding, which showed that autogenous healing performance improves at higher replacement ratios of GGBFS [21,40]. Since the cracking of the specimen was induced at the age of seven days, the OPC also had a large amount of unreacted binder, and the reaction of GGBFS did not occur sufficiently because of the short self-healing period of seven days, which is shorter than that observed in other studies [21,40]. The reaction of FA started after the generation of Ca(OH)_2_ from the reaction of OPC [41]. Thus, it seems that the reaction of GGBFS did not occur because there was an insufficient amount of Ca(OH)_2_ inside the cracks. In the case of the specimen that contained both SF and CSA, the self-healing product content was similar to that of P100. By contrast, in the cases of G40NS5, C10A2NS3, and G40C7NS5NC3, which contained CAs, the self-healing product content increased compared to P100. G40NS5 exhibited a self-healing product content approximately twice that of G40. This was because Na_2_SO_4_ promoted the reaction of OPC and GGBFS [42]. In the cases of C10A2NS3 and G40C7NS5NC3, the self-healing products appear to have increased because anhydrite and Na_2_SO_4_ promoted the reaction.

### 3.2. XRD Analysis

**Figure 6:**
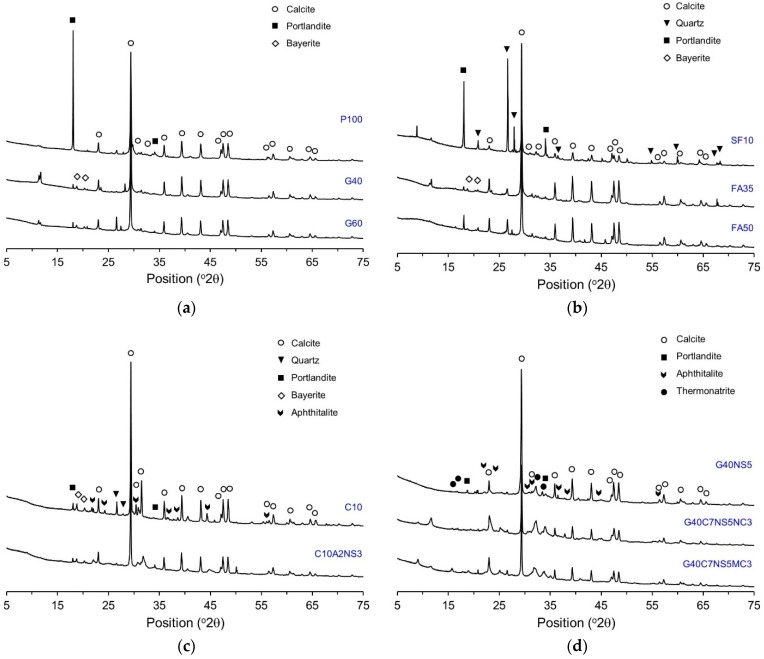
XRD patterns of self-healing products. (**a**) P100, G40, G60. (**b**) SF10, FA35, FA50. (**c**) C10, C10A2NS3. (**d**) G40NS5, G40C7NS5NC3, G40C7NS5MC3.

Figure 6 shows the XRD patterns of the self-healing products. In the case of P100, peaks of calcite and portlandite mainly appeared. It seems that portlandite was generated with the further hydration of OPC, and calcite with the further hydration of OPC and the reaction between Ca^2+^ and CO_3_^2−^ from the dissolution of CO_2_. In the case of G40 and G60, the peak of portlandite decreased, and peaks of calcite and bayerite were observed. The peak of portlandite decreased because portlandite was consumed in the reaction of the unreacted GGBFS. For SF10, quartz appeared in addition to calcite and portlandite. The quartz seems to have originated from SF. As with G40 and G60, the peak of portlandite decreased in FA35 and FA50, and the peak of bayerite appeared. In the case of C10 and C10A2NS3, the peak of calcite was observed, with some peaks of quartz, portlandite, bayerite, and aphthitalite. Aphthitalite was likely generated due to the anhydrite in the CSA. Similar to G40, G40NS5 exhibited a small portlandite peak, whereas aphthitalite and thermonatrite were generated by Na_2_SO_4_.

Table 3 lists the quantitative analysis results of the crystal phase of the self-healing products obtained by Rietveld refinement based on the XRD pattern experimental results of each specimen. In this table, amorphous means a hydrate that is not crystalline. The main components of the self-healing products generated from P100 were calcite and portlandite, with proportions of 33% and 23%, respectively. In the case of G40 and G60, bayerite appeared in addition to calcite and portlandite. With an increase in the mixture proportion of GGBFS, the portlandite content decreased, because portlandite was consumed in the hydration of GGBFS. The proportions of calcite in the G40 and G60 were 37% and 32%, respectively, similar to P100. As with P100, the main components of SF10, F35, and F50 were calcite and portlandite. In the case of SF10, the proportions of calcite and portlandite were 29% and 17%, respectively, a slight decrease compared to P100, but not a large difference. As for F35, the proportion of calcite was 32%, similar to that of P100; however, the proportion of portlandite decreased to 11% because the portlandite was consumed in the Pozzolan reaction of FA. In the case of F50, both the calcite and portlandite contents decreased compared to P100. This was likely because the proportion of portlandite and the pH were insufficient for the reaction of FA.

In the case of G40NS5 to which Na_2_SO_4_ was added, the proportion of portlandite decreased compared to G40, while aphthitalite, a type of alkali sulfate, and thermonatrite, a type of alkali carbonate, were generated. It seems that aphthitalite and thermonatrite were generated because Na^2+^, which was dissolved from Na_2_SO_4_, flowed through the cracks and reacted with SO_4_^2−^ and CO_3_^2−^. In the C10 and C10A2NS3, which contained a CSA expansion agent, the proportion of portlandite decreased the most, while aphthitalite and bayerite were generated. C10 exhibited the highest proportion of calcite at 39%, while containing 9% aphthitalite and 3% bayerite. In the case of C10A2NS3, the proportion of calcite was 24%, which was lower than that of P100, whereas the proportions of aphthitalite and bayerite were 8% and 2%, respectively. As for G40C7NS5NC3 and G40C7NS5MC3, the main components were calcite, aphthitalite, which is an alkali sulfate, and thermonatrite, which is an alkali carbonate. For G40C7NS5NC3, the proportion of aphthitalite was 18%, which was higher than the proportion of calcite. The quantitative analysis results of the self-healing products showed that calcite accounted for the highest proportion in every specimen, excluding G40C7NS5NC3. Therefore, calcite was concluded to be the main component in the self-healing products. An autogenous healing product varies with the type of mineral admixture used. When CAs were used, the results showed other types of compounds that did not appear in P100.

### 3.3. TG/DTG Analysis

In this study, the components of self-healing healing were analyzed using TG/DTG analysis. The decomposed materials of hydrates differ by temperature. The weight of C-S-H decreases because H_2_O evaporates at 200 °C or lower [43]. Bayerite (Al(OH)_3_) is decomposed in the temperature range of 180–250 °C [44]. For portlandite (CH), the decomposition of H_2_O occurs in the temperature range of 400–550 °C, and for calcite, the decomposition of CO_2_ occurs in the temperature range of 550–850 °C [45]. 

**Figure 7:**
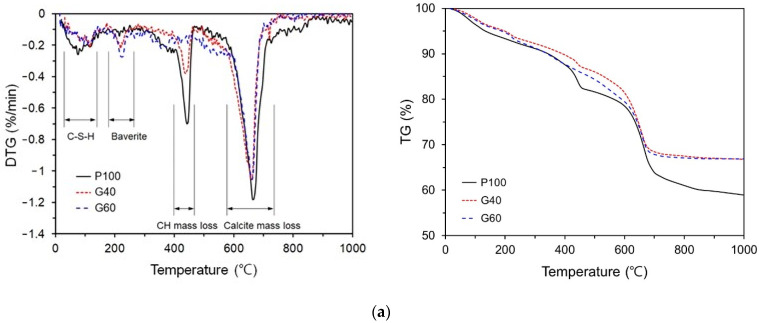

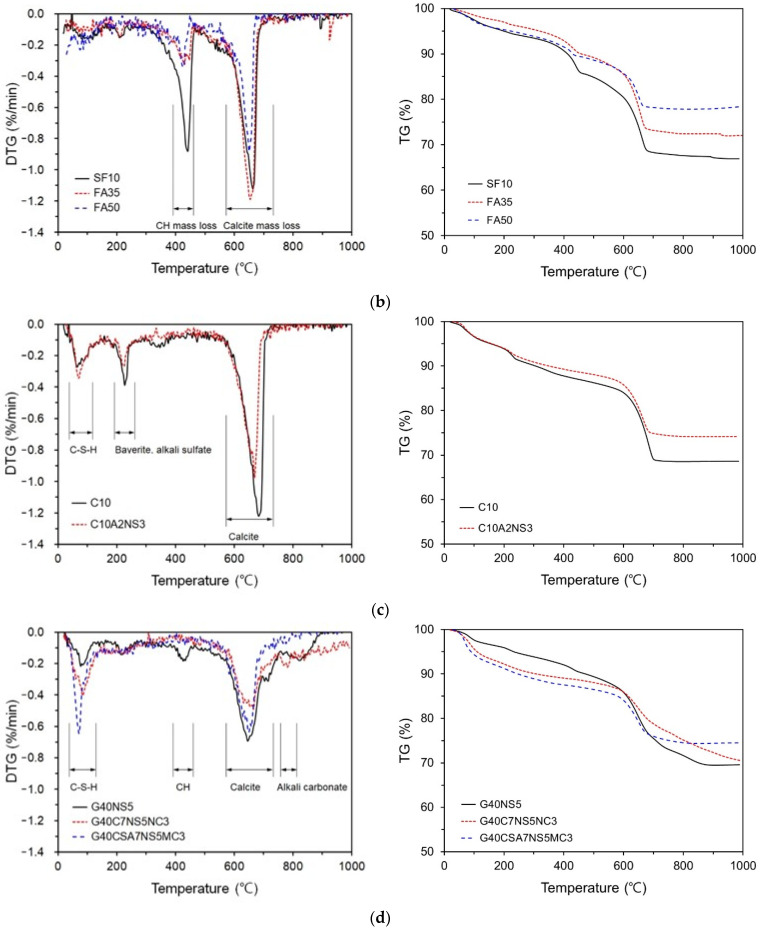
Thermal gravimetric analysis results of self-healing products. (**a**) P100, G40, G60. (**b**) SF10, FA35, FA50. (**c**) C10, C10A2NS3. (**d**) G40NS5, G40C7NS5NC3, G40C7NS5MC3.

Figure 7 shows the TG/DTG analysis results. In the case of P100, the weight changed because of the decompositions of C-S-H, CH, and calcite. The weight changes due to the decompositions of CH and calcite were particularly significant, indicating that the self-healing products contained large amounts of CH and calcite. In contrast to P100, G40 and G60 exhibited weight changes due to the decomposition of bayerite, and the weight change due to the decomposition of CH was lower than of P100. It appears as if GGBFS reacted with CH to form bayerite. G60 did not show any weight change due to the decomposition of CH. It appears that G60 does not form the CH required for GGBFS to react due to its low OPC. G40 and G60 showed significant weight changes due to the decomposition of calcite, albeit to a lower extent than in P100. In G40 and G60, the concentration of Ca^2+^ in the pores was smaller than that of OPC, so it seems that the generation of calcite decreased. There was no difference in the weight of calcite due to the replacement ratio of GGBFS. In the cases of F35 and F50, the weight changed because of the decompositions of CH and calcite. The CH content decreased significantly compared to that of P100 regardless of the mixture proportion of FA, because CH was consumed by the pozzolanic reaction of FA. The calcite content in F35 was similar to that in P100, whereas the calcite content in F50 decreased. SF10 and P100 exhibited similar shapes on the graph. When 10% silica fume was used, it did not yield any significant effect on the contents of C-S-H, calcite, and Ca(OH)_2_, which were present in the self-healing products.

In the cases of C10 and C10A2NS3, the weight changed because of the decompositions of C-S-H, bayerite, alkali sulfate, and calcite. The weight change due to the decomposition of C-S-H was similar to that in the case of P100. Results of XRD/Rietveld refinement analysis also show alkali sulfate in C10 and C10A2NS3. Figure 7d shows the thermal gravimetric analysis results of G40NS5, G40C7NS5NC3, and G40C7NS5MC3. The graphs show that the weight changes of G40C7NS5NC3 and G40C7NS5MC3 due to the decomposition of C-S-H were greater than that of G40NS5. This section is where the weight change due to the decomposition of C-S-H generally appears, and G40C7NS5MC3 showed a significant weight change in this section. A weight change due to the decomposition of Ca(OH)_2_ appeared only in G40NS5, and it was similar to that of G40. The weight change due to the decomposition of calcite was lower than that of G40, and the weight change of G40C7NS5NC3 was the lowest. However, in the case of G40C7NS5NC3, a small weight change due to the decomposition of alkali carbonate was observed. The XRD analysis result also showed that G40C7NS5NC3 produced 3% alkali carbonate.

**Figure 8:**
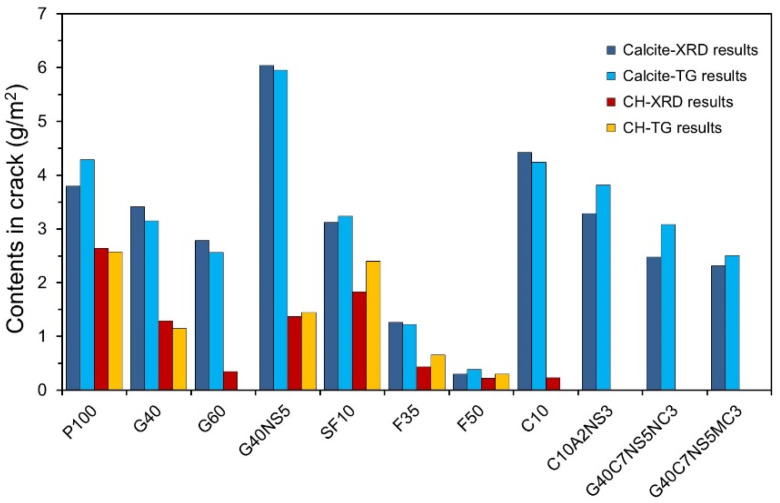
Calcite and portlandite contents of self-healing products.

Figure 8 shows the contents of calcite and CH measured from the XRD/Rietveld refinement and TG analyses. Y-axis indicates the contents of calcite and CH generated in cracks shown in units of amount per crack surface area (g/m^2^). The graph shows that the quantitative analysis results of XRD/Rietveld refinement and TG/DTG analysis were similar. Except for F35 and F50, the calcite content was higher by 2.3 g/m^2^ or more. In F35 and F50, the calcite contents were very low: 1.26 g/m^2^ and 0.3 g/m^2^, respectively. When a mineral admixture was added, the calcite content in the self-healing products was lower than in P100, except for G40NS5 and C10. In the case of G40NS5, the calcite content increased by more than 60%, which was likely because Na_2_SO_4_ promoted the reaction between OPC and GGBFS [42]. The portlandite content was highest in P100, and it decreased when the mineral admixture was added. In particular, portlandite did not appear in G60, C10, C10A2NS3, G40C7NS5NC3, and G40C7NS5MC3. Portlandite is a product of cement hydration, and its content decreases if OPC is replaced by another binder. Furthermore, GGBFS and FA produce a silicate gel by consuming portlandite, which seems to be the reason for the decrease in the portlandite content. Compared with previous findings that showed improvement in autogenous healing performance owing to the addition of SCMs and CAs, portlandite was not found to be advantageous for autogenous healing performance in our study [46,47].

Park and Choi [48] investigated self-healing potential in terms of the mixture proportions of OPC, GGBFS, FA, SF, CSA, and crystalline additives. After cracking, the hydration heat from the further hydration of unreacted binder was measured using isothermal calorimetry. It was predicted that the higher the hydration heat, the greater the amount of self-healing products generated. In the case of GGBFS, it was predicted that the higher the replacement ratio, the more the self-healing products due to further hydration. This result was different from that shown in Figure 8, where P100 is found to contain more self-healing products than G40 and G60. Figure 8 only shows the results of the calcite and portlandite, with no data pertaining to the quantitative analysis results of other phase compositions. Qiu et al. [49] reported the existence of C-S-H in the self-healing products of specimens containing GGBFS. Since a self-healing product is composed of various phase compositions, it is difficult to evaluate autogenous healing performance only by a quantitative analysis of the calcite and portlandite. Park and Choi [48] found that the higher the replacement ratio of FA, the lower the hydration heat, and the self-healing products were expected to decrease. This is similar to the result shown in Figure 8, and it is expected to improve the autogenous healing performance. However, the findings of some studies differed from ours. Tittelboom et al. [21] reported that the healing performance improved when the replacement ratio of FA was 35% based on a crack closing test. In addition, Park and Choi [47] reported that replacing FA increased the autogenous healing performance compared to the plain case based on a water flow test. These findings suggest that the autogenous healing performance is affected by factors other than the phase compositions and volume of the self-healing products. Roig-Flores et al. [25] reported that the autogenous healing performance was affected by the crack geometry. Park and Choi [48] examined the self-healing potential for mixtures with 10% replacements of SF and CSA. In the case of C10 in Figure 8, the calcite content increased compared to P100; however, the portlandite content decreased, and the calcite and portlandite contents of SF10 also decreased. Therefore, it can be concluded that phases other than calcite and portlandite were generated.

### 3.4. SEM–EDS Analysis

**Figure 9:**
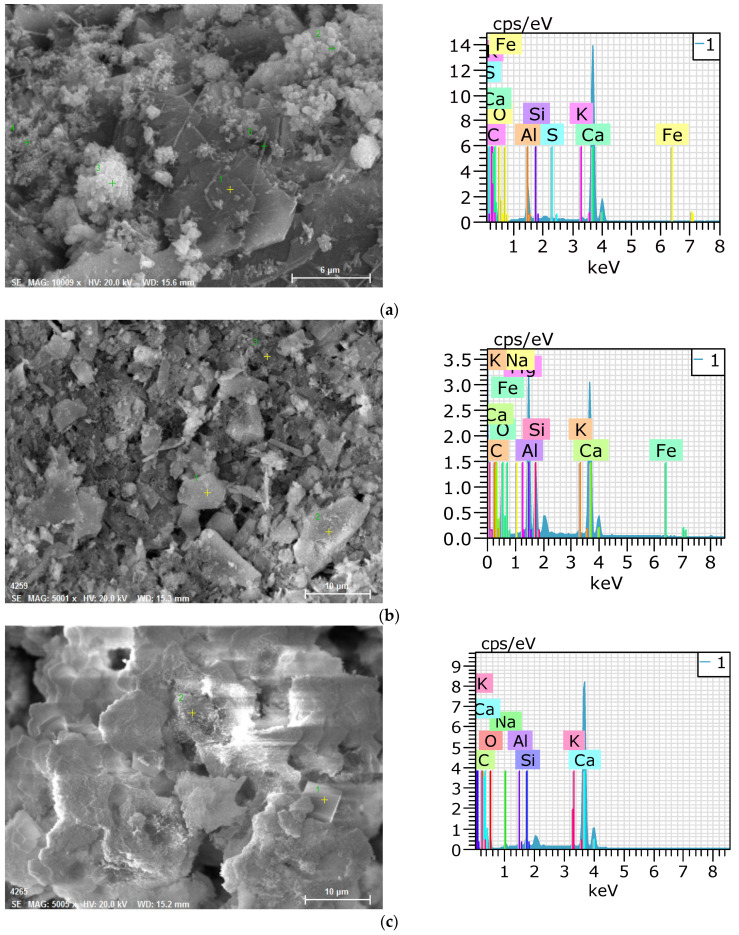

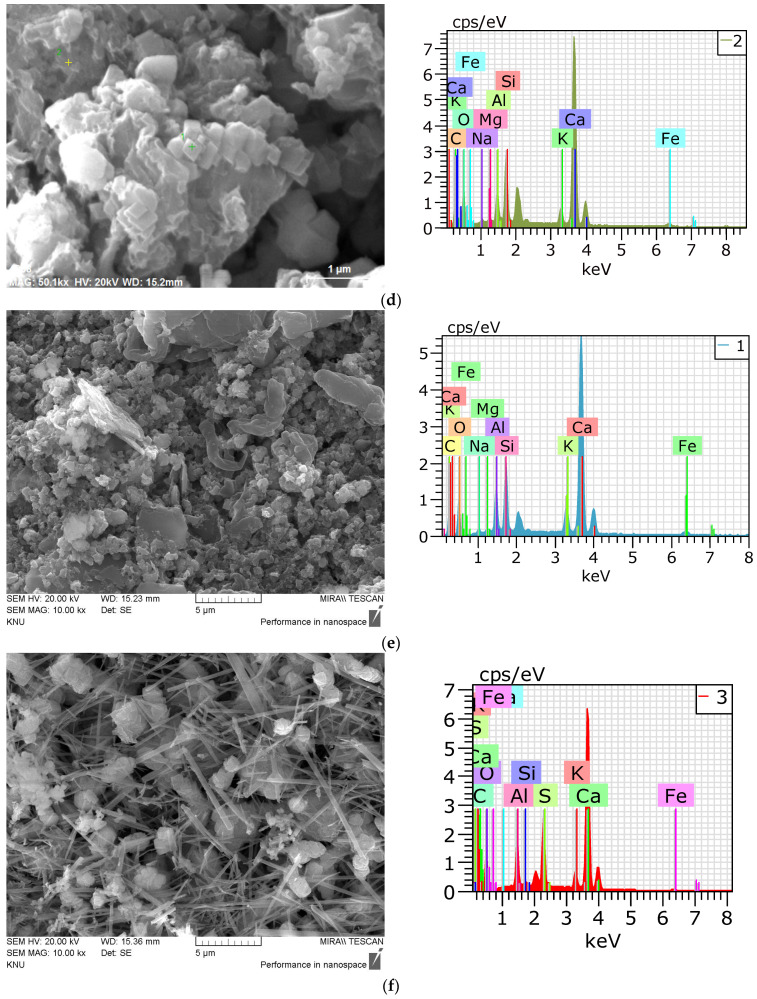

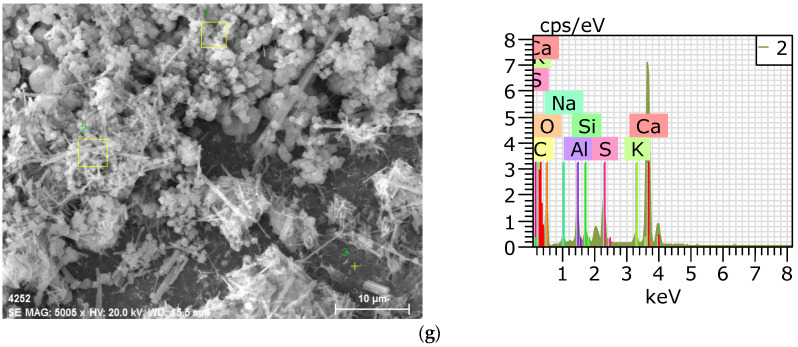
SEM-EDS observation results of self-healing products. (**a**) P100. (**b**) G40. (**c**) G40N5. (**d**) SF10. (**e**) F35. (**f**) C10. (**g**) C10A2NS3.

In this study, the morphology and chemical composition of self-healing products were investigated using SEM-EDS analysis. Figure 9 shows the SEM images and EDS analysis results of the self-healing products. The self-healing products of P100 mainly comprise plate-type hydrates and hexahedral hydrates. The EDS analysis result showed particularly high peaks for Ca, C, and O, and the hydrates were portlandite and calcite. G40 did not exhibit plate-type hydrates, and only some hexahedral hydrates appeared. The EDS analysis result showed high peaks for Ca, Si, Al, and O. Based on the EDS analysis result, it can be inferred that the major hydrate is C-S-H or C-A-H. The SEM image of G40N5 mainly showed hexahedral hydrates. The EDS analysis result indicated high peaks of Ca, C, and O, suggesting the dominant presence of calcite. The XRD/Rietveld refinement and TG/DTG analysis results yielded the same finding. The SEM image of SF10 mainly showed hexahedral hydrates. The EDS analysis result showed peaks for Ca, Si, C, and O. This confirmed that calcite and C-S-H were mixed in the autogenous healing product. In the EDS analysis result of F35, the peaks of Ca, Al, and Si were high, much like the case of G40. Thus, the self-healing products were concluded to be C-S-H and C-A-H. C10 showed needle-like hydrates, and the peaks of Ca, Al, S, and O mainly appeared in the EDS analysis result. Therefore, it is concluded that the autogenous healing product of C10 contains ettringite. C10A2NS3 mainly exhibited needle-like and hexahedral hydrates. The EDS analysis result also showed high peaks of Ca, Al, S, O, and C, suggesting the presence of ettringite and calcite in the self-healing products. The SEM–EDS analysis of the self-healing products generated by C10 and C10A2NS3 showed that when CSA is used, the self-healing products could contain ettringite, which is an expandable hydrate.

## 4. Conclusions

This study investigated the self-healing products of cement pastes mixed with SCMs and CAs. The self-healing products were quantified through XRD/Rietveld refinement and TG/DTG analysis, and the morphology and phase composition were analyzed using SEM–EDS. The findings of this study can be summarized as follows.

(1) The amount of self-healing products decreased when SCMs were used, whereas it increased when SCMs were used along with CAs. G40NS5 showed the highest content of self-healing products.
(2) The XRD/Rietveld refinement of the phase composition of the self-healing products showed that calcite was the main compound, irrespective of the mixture proportions. The portlandite content decreased when SCMs and crystalline additives were mixed. When CAs were mixed, alkali sulfate and alkali carbonate appeared. This indicates that the phase composition of self-healing products varies with the mixture proportions.
(3) The TG/DTG analysis results showed that the addition of GGBFS and FA significantly decreased the portlandite content in the self-healing products. In every specimen, the weight change due to the decomposition of calcite was the highest, and the calcite content decreased in the G40NS5, G40C7NS5NC3, and G40C7NS5MC3 with crystalline additives.
(4) The quantitative analysis results of calcite and portlandite obtained using the XRD/Rietveld refinement and TG/DTG analysis methods were compared with the results of existing studies. The calcite and portlandite contents were not found to be proportional to the autogenous healing performance. This is believed to be due to the effect of different types of phase compositions in the self-healing products and the crack geometry.
(5) The SEM–EDS analysis results showed that portlandite and calcite were mainly observed in P100 and SF10. When SF was replaced with 10%, the self-healing products were similar to the case using OPC alone. When GGBFS and FA were added, portlandite did not appear, and C-A-H and C-S-H mainly appeared. When the CSA was used, plate-type monosulfate and acicular ettringite appeared in the self-healing products, whereas plate-type and hexahedral hydrates mainly appeared in the G40NS5.

## Figures and Tables

**Table 1:** Chemical compositions and physical properties of raw materials.

	Chemical Compositions (wt. %)				
	OPC	GGBFS	FA	SF	CSA
CaO	63.13	45.2	5.24	0.17	3.6
SiO_2_	21.05	29.3	52.4	91.76	10.23
Al_2_O_3_	4.71	13.8	24.3	0.39	0.74
Fe_2_O_3_	3.23	0.53	6.96	0.9	53.74
MgO	3.06	4.13	1.46	1.23	0.78
K_2_O	1.67	0.45	1.56	0.97	0.2
Na_2_O	0.17	0.28	0.98	0.77	0.03
SO_3_	1.05	3.59	2.15	0.41	0.48
	Physical property				
Density (g/cm^3^)	3.14	2.9	2.15	2.35	2.73
Blaine fineness (m^2^/kg)	376	424	328	17,400 ^1^	708
Mean diameter (μm)	17.47	13.16	31.96	0.15	20.45

^1^ Specific surface measurements for silica fume taken using the nitrogen adsorption (BET) method.

**Table 2:** Chemical compositions and physical properties of raw materials.

	W/B	Binder (wt. %)					Crystalline Additive (wt. %)			
		OPC	GGBFS	FA	SF	CSA	Anhydrite	Na_2_SO_4_	Na_2_CO_3_	MgCO_3_
P100	0.4	100	-	-	-	-	-	-	-	-
G40	0.4	60	40	-	-	-	-	-	-	-
G60	0.4	40	60	-	-	-	-	-	-	-
G40NS5	0.4	55	40	-	-	-	-	5	-	-
SF10	0.4	90	-	-	10	-	-	-	-	-
F35	0.4	65	-	35	-	-	-	-	-	-
F50	0.4	50	-	50	-	-	-	-	-	-
C10	0.4	90	-	-	-	10	-	-	-	-
C10A2NS3	0.4	85	-	-	-	10	2	3		-
G40C7NS5NC3	0.4	45	40	-	-	7	-	5	3	-
G40C7NS5MC3	0.4	45	40	-	-	7	-	5	-	3

**Table 3:** Phase composition of self-healing products by Rietveld refinement.

	Self-Healing Products (wt. %)					
	Amorphous	Calcium Carbonate (CaCO_3_)	Portlandite (Ca(OH)_2_)	Alkali Sulfate (K,Na)_3_Na(SO_4_)_2_	Bayerite (Al(OH)_3_)	Alkali Carbonate (Na_2_CO_3_·H_2_O)
P100	44	33	23	-	-	-
G40	47	37	14	-	2	-
G60	63	32	4	-	1	-
G40NS5	60	31	7	1	<1	1
SF10	54	29	17	-		-
F35	57	32	11	-		-
F50	79	12	9	-	-	-
C10	47	39	2	9	3	-
C10A2NS3	66	24	-	8	2	-
G40C7NS5NC3	63	16	-	18	-	3
G40C7NS5MC3	73	20	-	7	-	<1

## Data Availability

Not applicable.
